# The accuracy of gap and step-off measurements in acetabular fracture treatment

**DOI:** 10.1038/s41598-021-97837-9

**Published:** 2021-09-14

**Authors:** A. M. L. Meesters, K. ten Duis, J. Kraeima, H. Banierink, V. M. A. Stirler, P. C. R. Wouters, J. P. P. M. de Vries, M. J. H. Witjes, F. F. A. IJpma

**Affiliations:** 1 Department of Surgery, University of Groningen, University Medical Center Groningen, Groningen, The Netherlands; 2 3D Lab/Department of Oral and Maxillofacial Surgery, University of Groningen, University Medical Center Groningen, Groningen, The Netherlands

**Keywords:** Bone, Fracture repair

## Abstract

The assessment of gaps and steps in acetabular fractures is challenging. Data from various imaging techniques to enable accurate quantification of acetabular fracture displacement are limited. The aim of this study was to assess the accuracy of pelvic radiographs, intraoperative fluoroscopy, and computed tomography (CT) in detecting gaps and step-offs in acetabular fractures. Sixty patients, surgically treated for acetabular fractures, were included. Five observers (5400 measurements) measured the gaps and step-offs on radiographs and CT scans. Intraoperative fluoroscopy images were reassessed for the presence of gaps and/or step-offs. Preoperatively, 25% of the gaps and 40% of the step-offs were undetected on radiographs compared to CT. Postoperatively, 52% of the gaps and 80% of the step-offs were missed on radiographs compared to CT. Radiograph analysis led to a significantly smaller gap and step-off compared to the CT measurements, an underestimation by a factor of two. Approximately 70% of the residual gaps and step-offs was not detected using intraoperative fluoroscopy. Gaps and step-offs that exceed the critical cut-off indicating worse prognosis often remained undetected on radiographs compared to CT scans. Less-experienced observers tend to overestimate gaps and step-offs compared to the more-experienced observers. In acetabular fracture treatment, gaps and step-offs were often undetected and underestimated on radiographs and intraoperative fluoroscopy in comparison with CT scans. This means that CT is superior to radiographs in detecting acetabular fracture displacement, which is clinically relevant for patient counselling regarding treatment decisions and prognosis.

## Introduction

Pelvic radiographs and computed tomography (CT) scans are used to assess the fracture pattern and to determine the amount of displacement in acetabular fractures. Fracture gap and step-off measurements aid in the decision-making process regarding treatment strategy and preoperative planning. Limited data is available from direct comparisons of radiographs and CT scans and their ability in detecting fracture displacements^1–5^. Intraoperative fluoroscopy is used to evaluate whether the fracture fragments have been adequately reduced, however it is unknown how accurate this is and how much of the gap and step-off can be detected compared to radiographs and CT scans.

Traditionally, residual displacements are graded by Matta’s criteria^6^. The largest gap or step-off on postoperative radiographs determines the quality of the fracture reduction. Studies reporting acetabular fracture treatments routinely correlate the clinical outcome to the amount of residual displacement^2–4,6–9^. These studies either use radiographs or CT scans to detect gaps and step-offs. Controversy exists about using CT scans for the postoperative evaluation of acetabular fractures, due to higher radiation exposure and higher costs^4,10^. Nevertheless, postoperative CT scans are increasingly being performed to assess the residual displacement and screw positions^1–3,11,12^. Therefore, this study uses CT scans as a reference. Also, a standardised CT-based measurement method was recently introduced enabling consistent determination of residual displacement^13^. Verbeek et al.^13^ concluded that their standardised method is reliable for the assessment of reductions and that CT scans revealed worse reduction compared to radiographs.

Understanding the accuracy and limitations of the imaging modalities assists in the interpretation of studies reporting functional outcomes after acetabular fracture surgery: Can we accurately predict hip survivorship and the patient’s rehabilitation process when different imaging modalities are still being used? We hypothesized that radiographs and intraoperative fluoroscopy imaging underestimate the extent of the fracture displacement, but the degree of underestimation was still unknown. Thus, the aim of this study was to assess the accuracy of radiographs and intraoperative fluoroscopy compared to CT scans in detecting gaps and step-offs in acetabular fractures.

## Materials and methods

### Patients

All the patients in the pelvic registry (N = 256) who had suffered an acetabular fracture between 2007 and 2018, and were operated on using open reduction and internal fixation in our academic level one trauma centre (N = 138), were reviewed. Cases were included if a complete data set was available with pre- and postoperative radiographs and CT scans as well as intraoperative fluoroscopy images. Bilateral acetabular fractures with concomitant pelvic ring injury (N = 2) and patients with skeletal immaturity (N = 16) were excluded. The baseline characteristics were retrieved from the electronic patient files and all fractures were re-classified according to the AO/OTA and Letournel & Judet classification systems^14–16^. All patients were approached by posted mail or telephone and asked if they had a conversion to total hip arthroplasty (THA) at follow-up. Our study was performed in line with the principles of the Declaration of Helsinki. It was reviewed and a waiver (no. 2016.385) was provided by the institutional Medical Ethics Review Committee of the University Medical Center Groningen. Exemption was provided by the Ethics Committee regarding obtaining informed consent, in accordance with the Dutch law that this research does not qualify as medical research with humans.

### Imaging assessment

All the measurements were performed by two trauma surgeons with > 5 years of experience in pelvic surgery, two trauma surgeons with < 5 years of experience in pelvic surgery and one PhD candidate in pelvic surgery. First, the maximum gap and step-off were measured on the radiographs (the anteroposterior, obturator oblique or iliac view; standard Judet views). Second, the final intraoperative fluoroscopy images were assessed for the presence of a gap and/or step-off in any of the standard Judet views. Finally, the maximum fracture gap and step-off were measured on axial, coronal and sagittal CT slices, similar to the method introduced by Verbeek et al.^13^. Each median gap or step-off measurement, determined by all five observers, was used to compare the measurements between the different imaging modalities. All CT scans had a maximum slice thickness of 2 mm. The residual displacement on the postoperative images was graded according to Matta’s criteria^6^. Particular emphasis was placed on the evaluation of the imaging techniques and not on the results of the surgical treatment.

### Statistical analysis

The radiographs’ measurements were compared to CT measurements with the Wilcoxon signed rank test (in SPSS version 23, IBM, Chicago, IL, US). The sensitivity, specificity, positive predictive value (PPV) and negative predictive value (NPV) of detecting gaps and step-offs using radiographs and intraoperative fluoroscopy was calculated using the CT measurements as a reference. Furthermore, the radiological findings (median postoperative gap and step-off sizes) were correlated with the clinical outcomes (conversion to THA) by using a Wilcoxon signed rank test. For patients with or without a THA, the quality of the fracture reduction was assessed on both postoperative radiographs and CT scans. A postoperative gap of ≥ 5 mm and/or a step-off of ≥ 1 mm was considered an inadequate reduction according to the criteria of Verbeek et al.^17^ For both radiographs and CT scans, the ability to correlate inadequate fracture reduction to conversion to THA was assessed by using descriptive statistics. Finally, gap and step-off measurements of more- compared to less-experienced observers were assessed by using a Wilcoxon signed rank test. A p-value of ≤ 0.05 was considered significant.

## Results

### Patients

Sixty patients with acetabular fractures, treated with open reduction and internal fixation, were included. The patient characteristics are presented in Table 1. Twelve out of 60 patients (20%) received a THA after a mean follow-up of 25 ± 22 months. One patient died within a month after the accident.

**Table 1:** Patients’ demographics.

Patient demographics (N = 60)	
**Sex (no.)**	
Male	51
Female	9
**Mean age (in years) (range)**	49 (19–81)
**Classification AO/OTA (no.)**	
A	20
B	19
C	21
**Classification Letournel & Judet (no.)**	
Elementary fracture types	16
Posterior wall	12
Anterior column	3
Transverse	1
Associated fracture types	44
Posterior column and wall	4
Transverse and posterior wall	10
T-shaped	4
Anterior column/wall with posterior hemitransverse	4
Both column	22

### Preoperative imaging

**Figure 1:**
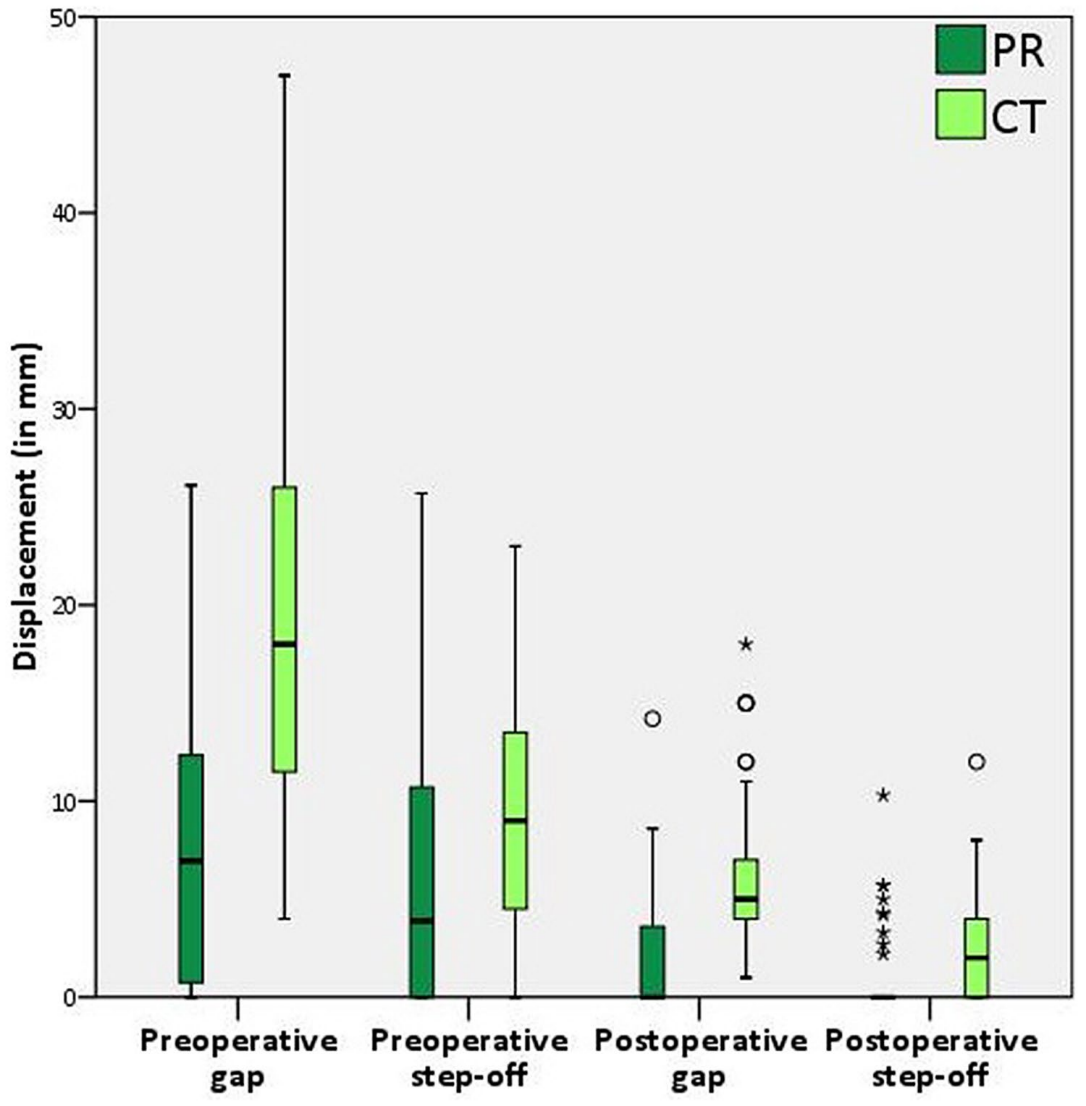
A boxplot comparing the pre- and postoperative gap and step-off measurements on radiographs (PR, in dark green) and CT scans (in light green).

**Figure 2:**
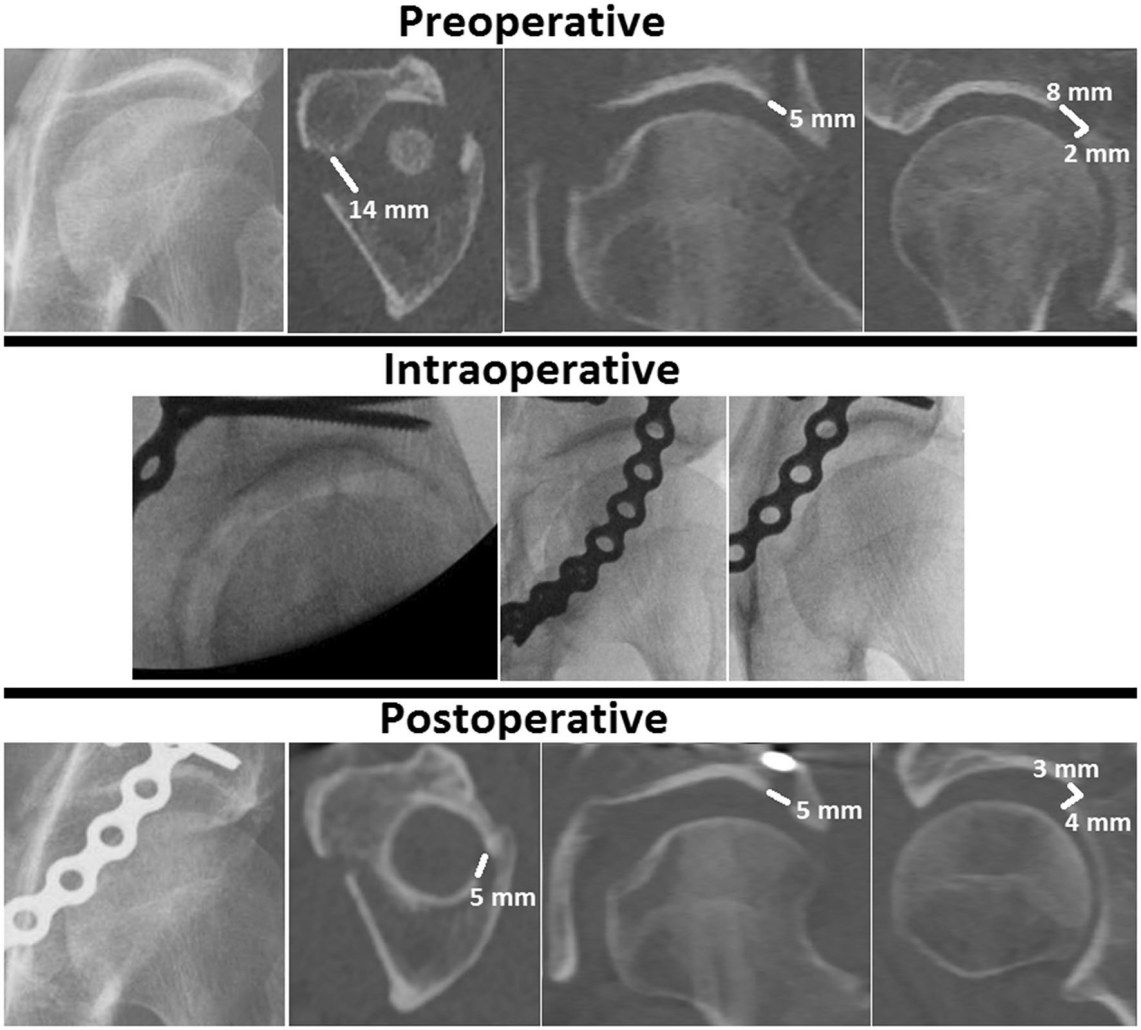
Case example of a both column (AO/OTA 62 C) fracture showing the discrepancy between radiograph, CT scan and intraoperative fluoroscopy images. No gap or step-off was observed on the radiographs and intraoperative fluoroscopy images, whereas the CT images demonstrated gaps and a step-off in all three planes (in white). The preoperative gap was 14 mm on the axial CT slice, 5 mm on the coronal CT slice and 8 mm on the sagittal CT slice. The preoperative step-off was 2 mm, measured on the sagittal CT slice. Postoperatively, a gap of 5 mm (axial and coronal) and 3 mm (sagittal) was measured and the step-off was 4 mm (sagittal).

A gap was observed on 45 patients’ preoperative radiographs, while a gap was present on the preoperative CT scan in all 60 patients. Radiograph analysis led to a significantly smaller gap compared to the CT measurements (P < 0.001), an underestimation of the gap by a factor of two (Fig. 1). The median size of the undetected gaps was 15 mm (IQR 10–23 mm) (Table 2). The sensitivity, specificity, PPV and NPV are presented in Table 2. Figure 2 shows a case example where no displacement was observed on the radiographs and intraoperative fluoroscopy whereas the CT scan revealed that displacement was indeed present.

**Table 2:** The sensitivity, specificity, positive predictive value and negative predictive value of pelvic radiographs and intraoperative fluoroscopy in the detection of gaps and step-offs in acetabular fractures, with computed tomography as the gold standard.

Imaging and measurements			Sensitivity (%)	Specificity (%)	PPV^a^ (%)	NPV^b^	Undetected gap/step-off	
							Size (mm)	N
Pelvic radio-graphs	Gap	Pre^c^	75	0	100	0	0–10	5
							11–20	6
							> 20	4
		Post^d^	48	0	100	0	1–4	17
							> 5	14
	Step-off	Pre^c^	58	67	97	8	0–5	8
							6–10	3
							> 10	13
		Post^d^	21	100	100	33	> 1	34
Intraoperative fluoroscopy		Gap	100	0	30	n/a	1–4	18
							> 5	24
		Step-off	100	35	26	100	> 1	32

Additionally, the size and the number of the undetected gaps and step-offs on pelvic radiographs as well as fluoroscopy are provided. For the postoperative undetected gaps and step-offs the cut-off values presented by Verbeek et al. were used^18^.

^a^Positive predictive value.

^b^Negative predictive value.

^c^Preoperative.

^d^Postoperative.

A step-off was observed in 34 patients’ radiographs, while the CT scan revealed a step-off in 57 patients. The radiographs demonstrated a smaller median step-off (4 mm) than the CT scans (9 mm), meaning there was a tendency to underestimate the step-off by a factor of two (Fig. 1). The median size of the undetected steps was 12 mm (IQR 5–15 mm) (Table 2). In one case, a step-off was observed on the radiograph while the CT revealed a medially displaced quadrilateral plate instead of a step-off.

### Intraoperative assessment

A gap was observed in 18 patients using fluoroscopy, whereas the postoperative CT demonstrated a gap in all 60 patients. The median size of the gaps not detected by fluoroscopy was 5 mm (IQR 4–6 mm) on the corresponding CT images (Table 2). A step-off was observed in 11 patients using fluoroscopy, in contrast to 43 patients on the postoperative CT. The median size of the undetected steps using fluoroscopy was 3 mm (IQR 2–4 mm) (Table 2).

### Postoperative evaluation

**Figure 3:**
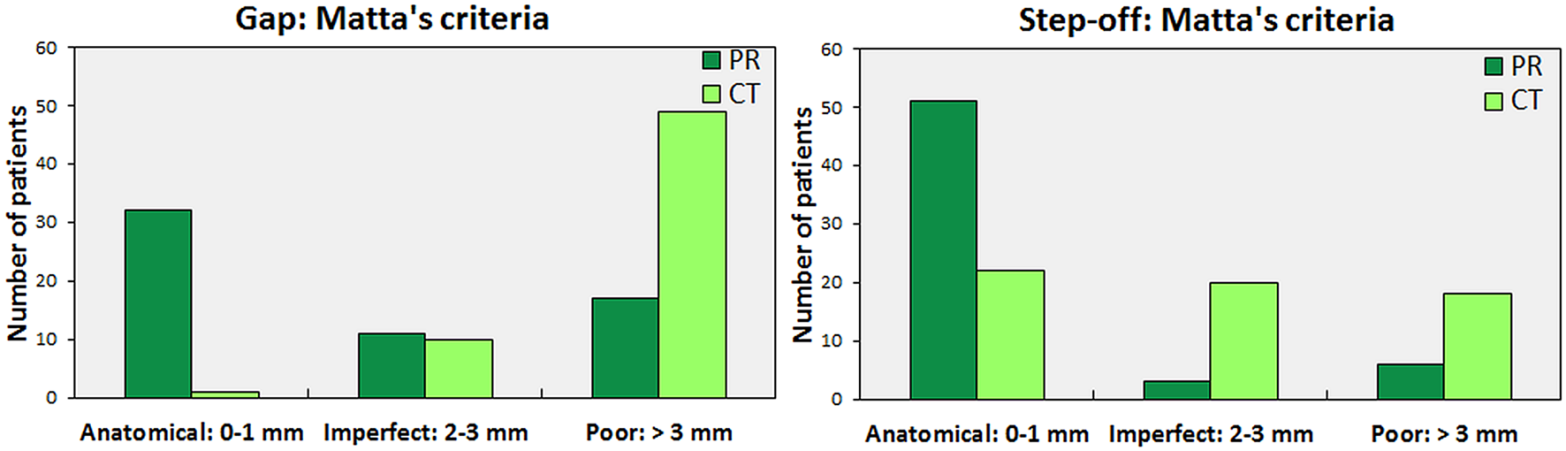
Grading of the residual gap (**a**) and step-off (**b**) on the postoperative radiographs (dark green) in comparison to CT scans (light green), according to Matta’s criteria.

A gap was only observed in 29 patients’ postoperative radiographs, while a gap was present in all 60 patients’ CT scans. The radiographs demonstrated a significantly smaller median gap compared to the CT measurements (P < 0.001) (Fig. 1). The median size of the undetected gaps was 4 mm (IQR 3–6 mm) (Table 2). Compared to the radiographs, CT showed worse reduction in 41 patients, the same quality of reduction in 18 patients, and better reduction in one patient (Fig. 3a). The patient with a better reduction on CT had a difficult to judge both column (62 C) fracture with multiple fracture lines.

A step-off was observed in nine patients’ radiographs, in contrast to 43 patients’ CTs. The median radiograph measurements of the step-offs were significantly smaller compared to the CT measurements (P = 0.048) (Fig. 1). The median size of the undetected steps was 3 mm (IQR 2–4 mm) (Table 2). Compared to the radiographs, the CTs showed worse reduction in 30 patients (50%), the same quality of reduction in 28 patients (47%), and better reduction in two patients (3%) (Fig. 3b). The postoperative CT results of two patients with a poor reduction due to a medially displaced quadrilateral plate turned out to be better than the radiograph estimate.

### Correlation with clinical outcomes

The median postoperative gap and/or step-off on both radiographs and CT scans was equal or larger for the patients that underwent a THA compared to those who retained their native hip at follow-up (Table 3). Radiographs tend to underestimate the sizes of the gap and step-off. For patients with a THA, three out of 12 patients (25.0%) had an inadequate reduction on the postoperative radiographs, compared to 11 patients (91.7%) on the postoperative CT scans, meaning that CT is superior to radiographs in correlating residual displacement to worse clinical outcome.

**Table 3:** Differences in median (interquartile range) postoperative gap and step-off on radiographs and CT scans between patients with a total hip arthroplasty (THA) and those who retained their native hip at follow-up.

Measurements	Imaging modality	THA (N = 12)	Native hip (N = 47)
Gap	Radiographs	2.4 (0.0–3.9) mm	0.0 (0.0–3.4) mm
	CT scans	6.0 (4.0–8.5) mm^a^	5.0 (4.0–7.0) mm^a^
Step-off	Radiographs	0.0 (0.0–0.0) mm	0.0 (0.0–0.0) mm
	CT scans	4.0 (2.3–5.0) mm^a^	2.0 (0.0–3.0) mm^a,b^

^a^Significant difference between measurements on radiographs and CT scans, with a P-value < 0.05.

^b^Significant difference in the median step-off on CT scans, between patients with a THA and those who retained their native hip at follow-up.

### Experience of the observers

Among less-experienced observers, the preoperative gap was larger on radiographs as well as on CT scans compared to more-experienced observers (Table 4). Less-experienced observers measured a larger preoperative step-off on radiographs whereas they found a smaller step-off on CT scans compared to the more-experienced observers. Moreover, less-experienced observers measured an equal or higher postoperative median gap or step-off on both radiographs and CT scans compared to the more-experienced observers.

**Table 4:** Differences in median (interquartile range) gap and step-off measurements between more-experienced and less-experienced observers.

	More-experienced observers	Less-experienced observers	P-value
**Preoperative gap**			
Radiographs	6.3 (1.9–10.7) mm	7.7 (1.9–14.2) mm	**0.003**
CT scans	17.5 (10.5–28.9) mm	19.5 (11.3–26.0) mm	0.080
**Preoperative step-off**			
Radiographs	6.3 (0.6–18.1) mm	3.7 (0–8.7) mm	**0.000**
CT scans	9.0 (3.5–13.9) mm	9.5 (5.3–15.0) mm	**0.024**
**Postoperative gap**			
Radiographs	0.0 (0.0–2.3) mm	2.9 (2.9–4.4) mm	**0.000**
CT scans	3.5 (2.1–5.5) mm	8.0 (6.0–10.0) mm	**0.000**
**Postoperative step-off**			
Radiographs	0.0 (0.0–0.0) mm	0.0 (0.0–0.0) mm	0.823
CT scans	1.0 (0.0–2.4) mm	4.0 (1.3–5.8) mm	**0.000**

P ≤ 0.05 was considered statistically significant.

## Discussion

This study demonstrated, that substantial gaps and step-offs often could not be detected or were underestimated on preoperative and postoperative radiographs compared to CT scans. Furthermore, a considerable number of the residual gaps and step-offs could not be observed using intraoperative fluoroscopy. A considerable number of patients with apparently limited displacement on radiographs do have substantial displacement according to the CT. Less-experienced observers tend to overestimate gaps and step-offs compared to the more-experienced observers. Gaps and step-offs that exceed the critical cut-off indicating worse prognosis often remained undetected on radiographs compared to CT scans. This means that CT is superior to radiographs in detecting fracture displacement, which is clinically relevant for patient counselling regarding treatment decisions or risks on conversion to THA.

The size of the initial displacement was underestimated by approximately a factor of two on the radiographs compared to CT scans. 25% of the patients’ gaps (with a median size of 15 mm) and 42% of the step-offs (with a median size of 12 mm) were missed on the radiographs. This emphasizes the limitation of diagnosing gaps and steps using radiographs. Accurate determination of the degree of the initial displacement is important since it affects the clinical outcome. Tannast et al.^18^ reported that a large initial displacement (≥ 20 mm) is associated with an increased risk of a conversion to total hip arthroplasty in the long term.

Intraoperative fluoroscopy could not detect approximately 70% of the residual gaps and step-offs compared to the postoperative CT scans. More than half of the undetected gaps were ≥ 5 mm, as measured on the postoperative CT scans, which is clinically relevant because a residual gap ≥ 5 mm is associated with in increased risk of conversion to total hip arthroplasty in the future^17^. To the best of our knowledge, only Norris et al.^19^ evaluated the use of and acknowledged the effectiveness of intraoperative fluoroscopy in acetabular fracture treatment in 1999. This is not consistent with our results, probably because the authors compared intraoperative fluoroscopy images with radiographs instead of CT images. Surgeons should be aware that residual gaps and steps can be easily missed or obscured by implants when using intraoperative fluoroscopy.

The quality of acetabular fracture reduction is determined by the residual displacement, and this parameter is routinely correlated with clinical outcome in the literature. In this study, the residual displacement on the radiographs of 28% of the patients showed a poor reduction according to Matta’s criteria. If one were to use the same criteria to assess the postoperative reduction on the CT scans, 82% of the patients would be judged as having a poor reduction. Therefore, Matta’s original radiographic-based criteria do not seem applicable for CT assessment of the fracture reduction. Verbeek et al.^2^ evaluated a large cohort of acetabular fractures and corroborated this with a mean residual gap of 3 mm on radiographs and 8 mm on CT scans. Furthermore, they found a mean residual step-off of 0 mm on radiographs and 2 mm on CT scans. They concluded that the residual displacement measured on CT scans is higher than that measured on radiographs. Our results support their observations.

**Figure 4:**
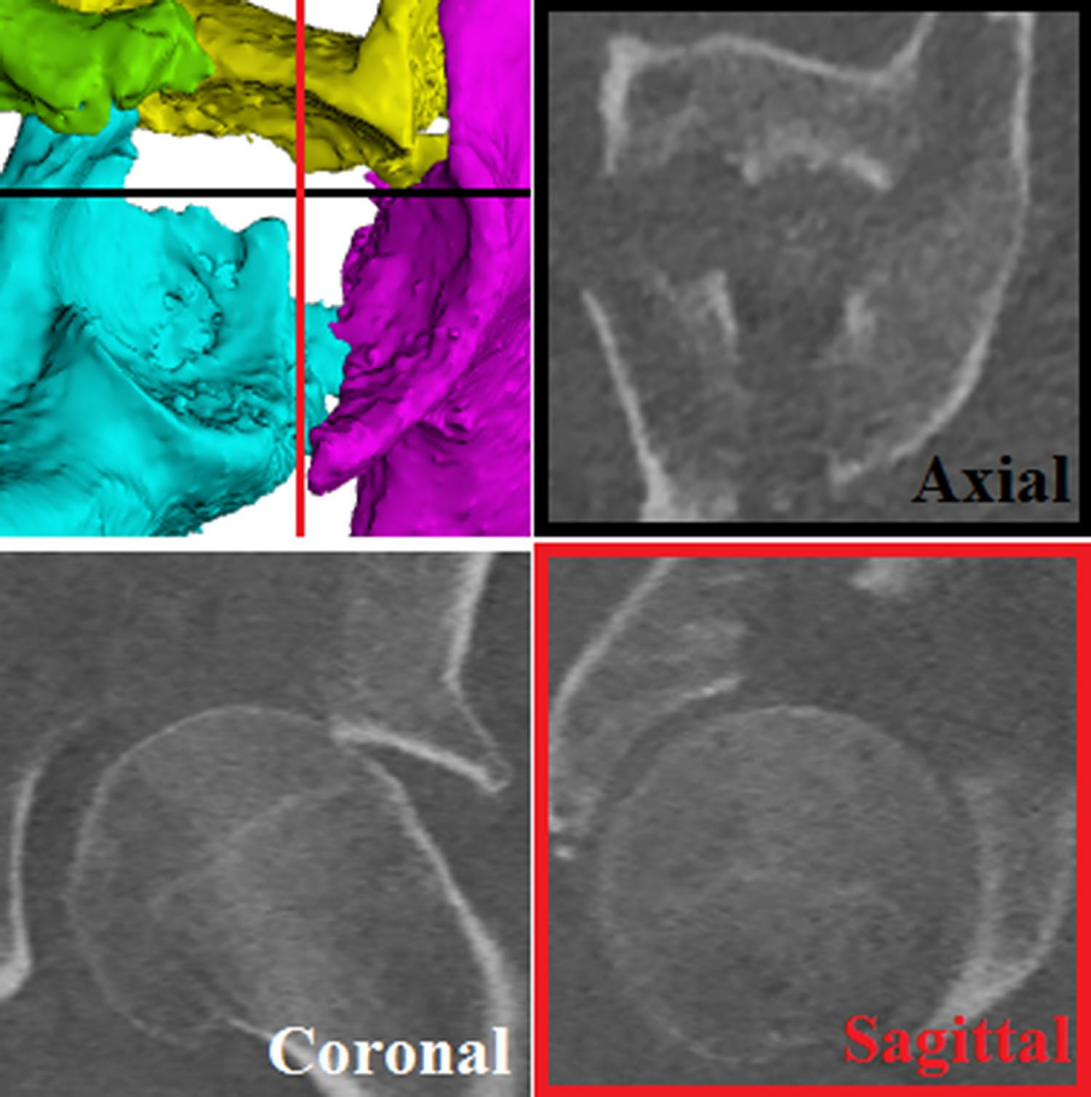
A gap and step-off can be seen on a 3D view of the acetabulum (top left). The black line represents the axial plane and the red line represents the sagittal plane. Multiple fracture lines, with gaps and step-offs, can be seen on the CT slices.

Radiographs appear to be inferior to CT scans in the detection rate and accuracy of gap and step-off measurements. This is mainly inherent to the imaging technique itself, because the three-dimensional spherical shape of the acetabulum is converted to a two-dimensional picture. This oversimplification will obscure fracture lines in different planes. Also, it is important to realize that the gap and step-off will be under- or overestimated when measuring at an oblique angle when a fracture line is not perpendicular to the CT plane it is measured in (Fig. 4). Although the difference in accuracy between a radiograph and a CT scan is small regarding simple elementary fractures, it seems to increase substantially for comminuted or associated fracture types. Moreover, metal implants will obscure parts of the fracture reduction on radiographs thus rendering it impossible to assess residual displacement in these areas. This is less of a restriction with CT scan metal artefact reduction techniques which can reveal more of the fracture reduction. Using radiographs to assess postoperative reduction will, most likely, lead to an underestimation of the residual gaps and step-offs, and subsequently a biased prognosis. This is supported by our findings that based on postoperative radiograph assessment only 25% of patients with a THA were considered as having an inadequate reduction, whereas after assessing the same patients on CT scans 92% turned out to have an inadequate reduction.

As to which fracture lines or which fracture fragments should be measured is, to date, arbitrary. For example, a both column (AO 62 C) fracture does not have a reference point because the acetabulum is disconnected from the rest of the pelvis. Additionally, it is questionable whether a single gap or step-off measurement is sufficient to assess the severity of the fracture and the quality of the reduction in complex fractures with multiple fracture lines^20^. Apart from the differences between measurements on various imaging modalities, the inter- and intra-observer reliability for gap and step-off measurements in acetabular fracture surgery was low as determined in our previous study^21^. Also, less-experienced observers tended to overestimate gaps and step-offs compared to the more-experienced observers.

The strengths of this study are that all measurements were performed by five independent observers, resulting in a total of 5400 measurements for the analysis. Also, to the best of our knowledge, this is the first study to analyse the entire perioperative imaging process for acetabular fracture treatment by using a standardised measurement technique^13^. The limitations of this study are that the intraoperative fluoroscopy images were not calibrated, therefore making it impossible to measure the size of the gap and step-off. Hence, the images could only be reassessed for the presence or absence of a gap or step-off. Additionally, some fracture types (e.g., posterior wall) were represented more frequently compared to others. Some fracture types might have been more difficult to measure than others, but the fracture types represented the distribution in our current practice and similar to the fracture types presented by Tannast et al.^18^.

With the advances in imaging modalities and hybrid operation theatres, intraoperative CT scans will probably be used more frequently to optimize intraoperative fracture visualization, reduction and fixation^22^. Intraoperative CT scans provide the opportunity to verify the reduction and implant positions during surgery and to perform immediate revisions if needed. Nonetheless, intraoperative CT is not available in all clinics. The assessment of the reduction, as presented in this study, applies to both intraoperative and postoperative CT scans. The decision for performing a postoperative CT scan should be left to the preference of the treating physician. In our perception, a postoperative CT scan could provide valuable information to evaluate operative results, improve individual surgical skills, evaluate innovative techniques, and inform patients about their prognosis. However, our studies demonstrated that 2D measurement techniques for acetabular fracture displacement have major shortcomings in terms of discrepancies in measurements on different imaging modalities and between observers^21^. In order to improve future results of acetabular fracture treatment, there is a demand for a reliable measurement technique that will consider potential 3D displacement of fracture fragments^20^. These measurements could be used in future clinical studies aiming to relate quantitative 3D CT measurements to treatment decisions and clinical outcomes. In line with these innovations, we envision that the use 3D fracture assessment, virtual surgical planning, and eventually personalized fracture treatment with patient-specific implants will be next steps in acetabular fracture treatment^20,23^.

In conclusion, clinicians should be aware of the differences between radiographs, fluoroscopy and CT scans in the ability to detect and estimate the size of gaps and step-offs in acetabular fracture treatment. Radiographs made at the time of the injury failed to reveal a quarter of the gaps, almost half of the step-offs, and underestimated the size of the gaps as well as the step-offs by a factor of two, compared to CT scans. Moreover, approximately three quarters of the residual gaps and step-offs could not be detected by final intraoperative fluoroscopy. Postoperative radiographs did not reveal half of the residual gaps, most of the step-offs, and significantly underestimated the amount of residual displacement compared to CT scans. Therefore, CT scans should be considered standard for optimal acetabular fracture assessment.

## Data Availability

The authors declare that the data supporting the findings of this study are available within the paper.
